# Complete section of the common bile duct during complicated cholecystectomy: laparoscopy-guided endoscopic treatment, a mini-invasive approach

**DOI:** 10.1055/a-2462-0801

**Published:** 2024-11-18

**Authors:** Pierre Mayer, Lucile Héroin, Guillaume Philouze, François Habersetzer, Patrick Pessaux, Abdenor Badaoui, Alfonso Lapergola

**Affiliations:** 1 36604 Gastroenterology and Hepatology, Les Hôpitaux Universitaires de Strasbourg, Strasbourg, France; 2 36604 Digestive and Endocrine Surgery, Les Hôpitaux Universitaires de Strasbourg, Strasbourg, France; 3 27083 Inserm U1110, Université de Strasbourg, Strasbourg, France; 4 82470 Department of Gastroenterology and Hepatology, CHU UCL Namur, Université catholique de Louvain, Yvoir, Belgium

Although widely practiced, cholecystectomy can expose patients to serious biliary injuries, including biliary leakage, stenosis, and more rarely section of the common bile duct (CBD) 1 2 .

We report here the case of a 29-year-old woman who had previously undergone sleeve gastrectomy. She presented with acute cholecystitis ( Fig. 1 ) and underwent a laparoscopic cholecystectomy. In the days following surgery, significant cholestasis became apparent, although without obvious symptoms. A computed tomography (CT) scan and magnetic resonance cholangiography did not reveal any major biliary lesions, but a collection was observed in the cholecystectomy site, with minor hepatic perfusion disorder.

**Fig. 1:**
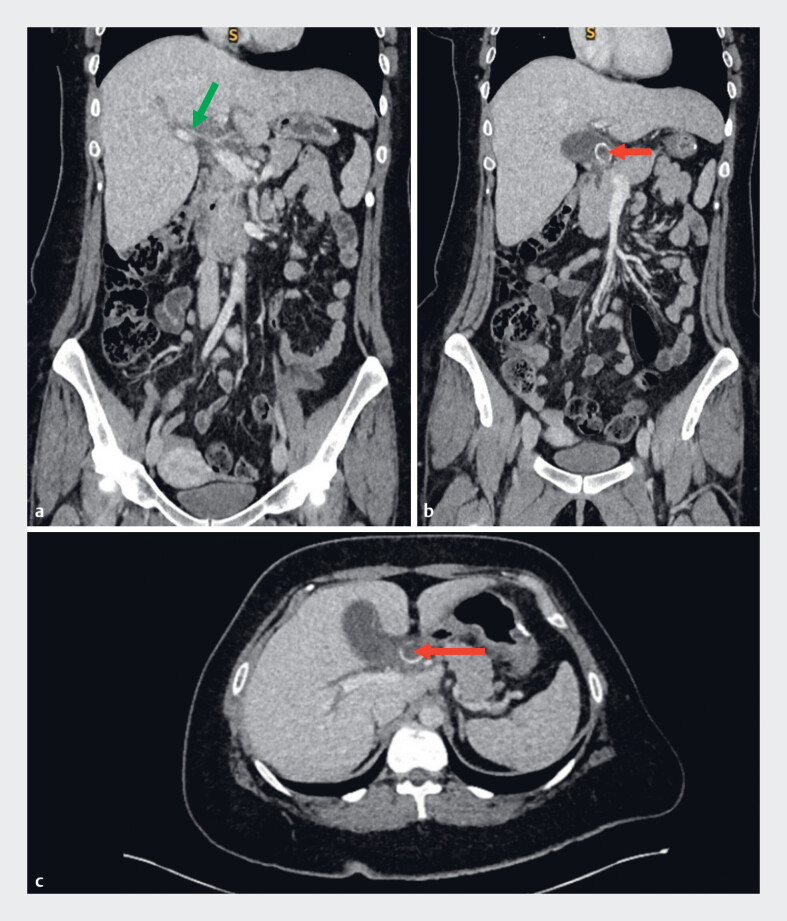
Abdominopelvic computed tomography scan before cholecystectomy showing: **a** on coronal section, minimal intrahepatic bile duct dilatation (green arrow); **b** on coronal section, cholecystitis with macrocalculi in the cystic duct (red arrow); **c** on axial section, cholecystitis with macrocalculi in the cystic duct (red arrow).

Subsequently, 10 days after the surgery, the patient’s condition remained unfavorable, with significant abdominal pain. A further CT scan was performed, which showed a large abdominal effusion, with fat infiltration in the cholecystectomy space ( Fig. 2 ). An emergency laparoscopy was performed, which revealed biliary peritonitis ( Fig. 3 ), so an endoscopic retrograde cholangiopancreatography (ERCP) was performed at the same time to treat the biliary leak ( Video 1 ).

**Fig. 2:**
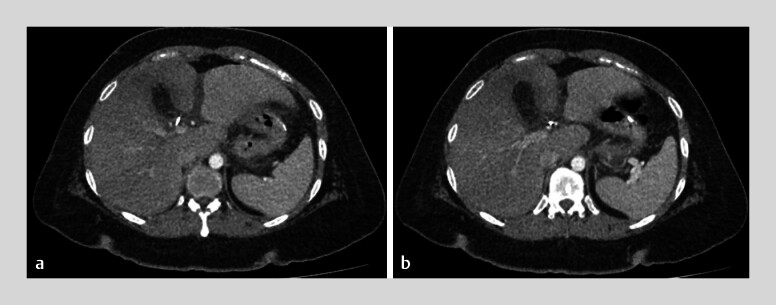
Abdominopelvic computed tomography scan 10 days after surgery showing on axial sections the cholecystectomy clips, with fat infiltration in the cholecystectomy site, but no bile duct dilatation.

**Fig. 3:**
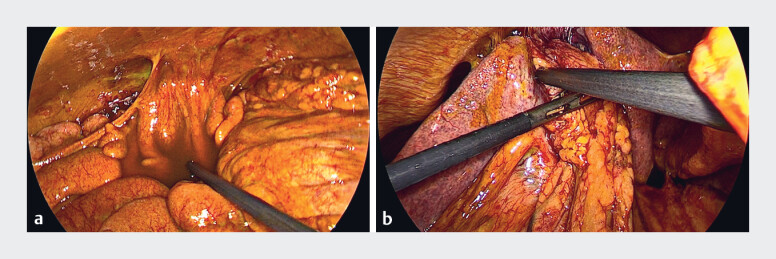
Laparoscopic image showing biliary peritonitis.

Endoscopic and laparoscopic repair of the common bile duct after it had been completely sectioned during laparoscopic cholecystectomy.Video 1

The laparoscopy revealed surgical clips closing the extrapancreatic CBD, which had been completely sectioned. All of the clips were removed, revealing two intrahepatic ducts, which were reached by ERCP under laparoscopic guidance. Biliary opacification revealed normal intrahepatic ducts ( Fig. 4 ), and two guidewires were positioned in the intrahepatic ducts and held in place surgically, which then allowed these ducts to be drained by placing 15- and 12-cm 7-Fr plastic stents ( Fig. 5 ). The left intrahepatic ducts could not be efficiently drained, but the cholangiogram showed that parts of these were draining into the right side of the liver. Additional percutaneous biliary drainage of the left side of the liver was performed to finalize the connection between the CBD and intrahepatic ducts and therefore facilitate the formation of a neo-CBD.

**Fig. 4:**
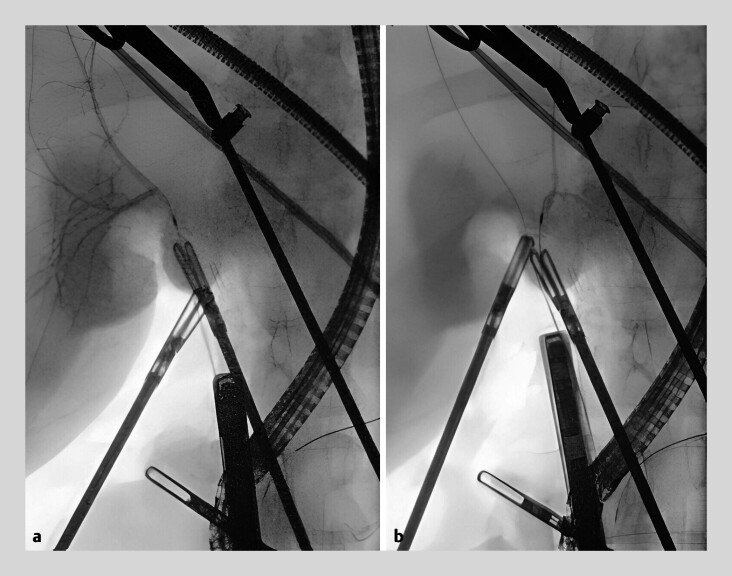
Fluoroscopic images showing bile duct opacification after laparoscopic- and endoscopic-guided selective cannulation.

**Fig. 5:**
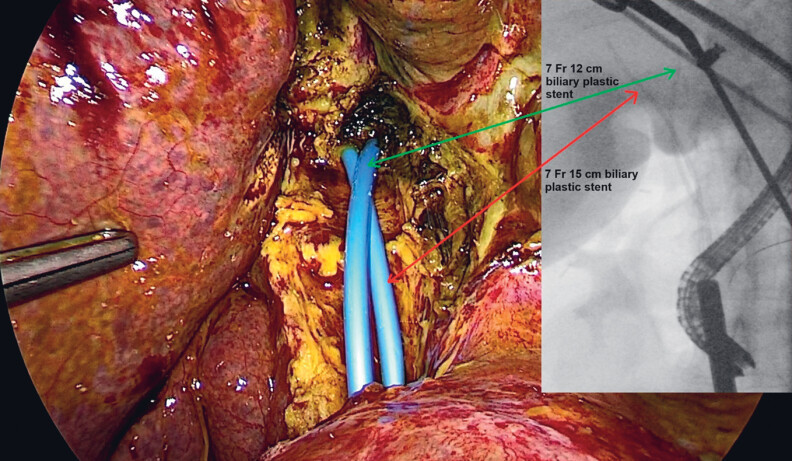
Laparoscopic and endoscopic view of the two plastic stents placed endoscopically to create a new common bile duct.

A few years ago, we illustrated the endoscopic treatment of a sectioned right posterior bile duct during laparoscopic cholecystectomy 3 . Here, we demonstrate the possibility of performing endoscopic treatment for a completely sectioned CBD under laparoscopic guidance, even at a distance from the biliary wound.

Endoscopy_UCTN_Code_TTT_1AR_2AG

## Notes

Endoscopy E-Videos https://eref.thieme.de/e-videos
*E-Videos* is an open access online section of the journal *Endoscopy* , reporting on interesting cases and new techniques in gastroenterological endoscopy. All papers include a high-quality video and are published with a Creative Commons CC-BY license. Endoscopy E-Videos qualify for HINARI discounts and waivers and eligibility is automatically checked during the submission process. We grant 100% waivers to articles whose corresponding authors are based in Group A countries and 50% waivers to those who are based in Group B countries as classified by Research4Life (see: https://www.research4life.org/access/eligibility/ ). This section has its own submission website at https://mc.manuscriptcentral.com/e-videos .

## References

[1] Flum DR Cheadle A Prela C Bile duct injury during cholecystectomy and survival in medicare beneficiaries JAMA 2003 290 2168 2173 14570952 10.1001/jama.290.16.2168

[2] Emara MH Ali RF Mahmoud R Postcholecystectomy biliary injuries: frequency, and role of early versus late endoscopic retrograde cholangiopancreatography Eur J Gastroenterol Hepatol 2021 33 662 669 10.1097/MEG.0000000000002086 33560689

[3] Mayer P Héroin L Habersetzer F Combined endoscopic and surgical management of a right intrahepatic bile duct injury during laparoscopic cholecystectomy Endoscopy 2022 54 E682 E683 35180791 10.1055/a-1743-1878

